# Mucosal MicroRNAs Expression Profiles before and after Exclusive Enteral Nutrition Therapy in Adult Patients with Crohn’s Disease

**DOI:** 10.3390/nu8080519

**Published:** 2016-08-22

**Authors:** Zhen Guo, Jianfeng Gong, Yi Li, Lili Gu, Lei Cao, Zhiming Wang, Weiming Zhu, Jieshou Li

**Affiliations:** Department of General Surgery, Jinling Hospital, Medical School of Nanjing University, No. 305 East Zhongshan Road, Nanjing 210002, China; guozhi0809@sina.com (Z.G.); gongjf03@126.com (J.G.); dryili@126.com (Y.L.); drliligu@126.com (L.G.); caolei.jinling@sina.com (L.C.); zhimingwangjl@sina.com (Z.W.); professorlijieshou@126.com (J.L.)

**Keywords:** Crohn’s disease, exclusive enteral nutrition, microRNAs expression

## Abstract

MicroRNAs (miRNAs) have been shown to be important for the pathogenesis of Crohn’s disease (CD). Exclusive enteral nutrition (EEN) is an effective therapy for inducing remission in CD. We aimed to investigate the alteration of miRNAs expression profile in the terminal ileal mucosa of CD patients before and after EEN. Twenty-five patients and ten healthy individuals were included. MiRNAs expression profile was firstly assessed using microarray technology and then validation was performed by qRT-PCR. The correlations between miRNAs and CD activity index (CDAI) score and serum C–reactive protein (CRP) level were also evaluated. Microarray analysis showed that mucosal miRNAs expression profile after EEN therapy was significantly changed compared with inflamed mucosa before treatment, and was most similar to the healthy one among all CD groups. Altered expressions of hsa-miR-192-5p, hsa-miR-423-3p, hsa-miR-99a-5p, hsa-miR-124-3p, hsa-miR-301a-5p, hsa-miR-495-5p, and hsa-let-7b-5p were confirmed by qRT-PCR. hsa-let-7b-5p was significantly correlated with serum CRP levels before and after EEN treatment (*r* = −0.518, *p* = 0.008, and *r* = −0.569, *p* = 0.003). Our study showed EEN induction therapy was associated with a trend for normalizing of the mucosal miRNAs expression profile, and expression of mucosal hsa-let-7b-5p was correlated with serum CRP level in patients with CD.

## 1. Introduction

Crohn’s disease (CD) is a chronic relapsing inflammatory bowel disorder that can affect any part of the gastrointestinal track. It is believed that the environment–gene interactions play a major role in the pathogenesis of CD [1,2] Epigenetic factors could mediate environment–gene interactions involved in pathogenesis, and microRNA (miRNA) is one of the main epigenetic mechanisms [3]. MiRNAs, the short (19–24 nucleotides) non-coding RNAs, are involved in many biological processes like cell differentiation, proliferation, and apoptosis by inhibiting translation or degrading the target mRNA [4].

MiRNAs play an important role in regulating intestinal barrier function and intestinal immune response. It has been demonstrated that some dysregulated miRNAs contribute to the abnormal inflammatory response in inflammatory bowel disease (IBD), and loss of intestinal miRNAs leads to damaged intestinal barrier function and inflammation, which is similar to IBD [5,6]. At the same time, cell type variations between samples and increased infiltration of inflammatory cells in CD may also lead to the different expression of certain miRNAs in serum and mucosa [7]. Therefore, these miRNAs expression profiles may be influenced by effective therapies.

Diet is one of the environmental factors closely relevant to CD. The incidence of CD has risen in countries with Western diet and several dietary factors such as protein, fat and sugar intake may contribute to the pathogenesis of CD [8]. Exclusive enteral nutrition (EEN) is one of the current treatments of CD. The benefits of EEN in CD were firstly reported in 1973 [9]. Since then, the efficacy of EEN on inducing remission and mucosal healing of CD has been confirmed by many studies, and EEN becomes a preferred therapeutic strategy for the treatment of CD in some medical centers [10,11,12,13,14]. However, the mechanism of EEN is still undiscovered.

It is now accepted that dietary components can interact with genome and modify certain types of gene expression under a normal and variable genetic status, which may become either risk or protective factors for certain diseases [15,16]. Evidences are emerging that a wide range of dietary factors (macronutrients, micronutrients and caloric restriction) can affect expressions of many miRNAs [17]. Our previous study has demonstrated both the inflamed and non-inflamed terminal ileal mucosa in active CD have their unique miRNAs expression profiles [18]. In this study, we compared the miRNAs expression profiles of the terminal ileal mucosa before and after EEN treatment in order to investigate the alteration of miRNAs expression profile in the terminal ileal mucosa before and after EEN, which may provide a new perspective for the mechanism of EEN in CD.

## 2. Materials and Methods

### 2.1. Patients

The study protocol was approved by the Institutional Ethics Committee of Jinling Hospital (Nanjing, China). All patients gave written informed consent (Ethical approval code No. 2011NLY-039).

Requirements for inclusion in the study were very strict to minimize the impact of potentially confounding factors like smoking, medication and surgery. Patients from Jinling Hospital satisfying the following criteria were recruited between August 2011 and June 2014: (1) patients within the age range of 17–40 years; (2) patients were diagnosed with ileal or ileocolonic CD using clinical, radiological, endoscopic and histological evidence; (3) patients had a Crohn’s disease activity index (CDAI) score of more than 150 and less than 400 and a serum C-reactive protein (CRP) level of more than10 mg/L at the enrollment; (4) patients had received no or stable treatment of sulfasalazine or mesalazine for more than one month; (5) patients had not received corticosteroids, immunosuppressive drugs, biological therapies or surgeries; and (6) patients had no history of smoking. Patients meeting the following criteria were excluded: (1) patients who presented with intestinal obstruction, fistula or abscess; and (2) patients who complicated with other immune associated diseases or systemic diseases. Normal healthy individuals undergoing screening ileocolonoscopies were also enrolled as the healthy control group.

### 2.2. Treatment

Patients were treated with EEN for at least 4 weeks. As previously described [19], a polymeric formula (Nutricia, Amsterdam, The Netherlands) was used in this study. During this period, any other food and drink except water was forbidden. The enteral nutritional suspension was infused continuously through a nasogastric tube. The daily calorie intake was 25–30 kcal/kg body weight. The dosage of EEN was gradually increased from one-half to the full strength over 2 days to reduce side effects. For patients who received stable treatment of sulfasalazine or mesalazine at enrollment, the dose remained unchanged during the entire study. Both clinical and hematological assessments were conducted to assess the efficacy of the treatment. Remission was defined as a CDAI score of less than 150 and the CRP level of less than 10 mg/L.

### 2.3. Intestinal Tissues Collection

For assays of miRNAs expression, three biopsy specimens per patient from either inflamed mucosa or from normal mucosa (>8 cm from the inflamed mucosa) were removed from terminal ileum at ileocolonoscopy 1–7 days before therapy. After treatment (1–7 days after EEN), patients who achieved clinical remission underwent a second ileocolonoscopy. Three biopsy specimens from the region where lesions were present before therapy were obtained. In addition, biopsy specimens were obtained from the terminal ileum of healthy individuals.

### 2.4. RNA Extraction

Total RNA including miRNAs of pinch biopsies were isolated immediately using TRIzol (Invitrogen) and miRNeasy mini kit (QIAGEN) according to manufacturer’s instructions. The RNA samples were stored at −80 °C until use in either the miRNA array analysis or quantitative reverse-transcription polymerase chain reaction (qRT-PCR). RNA quality, quantity and integrity were measured as previously described [18].

### 2.5. miRNA Microarray

In this study, the miRNAs expression profiles were examined using the 7th generation of miRCURYTM LNA Array (v.18.0) (Exiqon, Vedbaek, Denmark) covering all human miRNAs annotated in miRBase 18.0.

The miRCURY™ Hy3™/Hy5™ Power labeling kit (Exiqon, Vedbaek, Denmark) was used for miRNA labeling. Each sample was 3′-end-labeled with Hy3TM fluorescent label. Then, the Hy3TM-labeled samples were hybridized on the miRCURYTM LNA Array (v.18.0). After hybridization, the slides were achieved, washed using Wash buffer kit (Exiqon), and finally dried by centrifugation. Then the slides were scanned using the Axon GenePix 4000B microarray scanner (Axon Instruments, Foster City, CA, USA)., and grid alignment and data extraction were performed using GenePix Pro 6.0 software (Axon). The details were described in our previous study [17].

### 2.6. Quantitative Reverse-Transcription Polymerase Chain Reaction (qRT-PCR)

QRT-PCR was used to validate the expression of miRNAs. cDNA was generated from 800 ng of total RNA using miRNA-specific RT primers in Gene Amp PCR System 9700 (Thermo Fisher Scientific Inc., Waltham, MA, USA). QRT-PCR was then performed in a ABI PRISM7900 system (Thermo Fisher Scientific Inc., Waltham, MA, USA) at 95 °C for 10 min, followed by 40 cycles of 95 °C for 10 s and 60 °C for 60 s. Each sample was analyzed in triplicate. U6 was used as an endogenous control to normalize the expression level of target miRNAs. The relative expressions of miRNAs were measured using a comparative threshold cycle method (2^−ΔΔCT^). Primers are listed in Table 1.

### 2.7. Data Analysis

SPSS 17.0 (Chicago, IL, USA) and GraphPad Prism 5 (San Diego, CA, USA) were used to perform statistical analysis. Continuous data are expressed as mean ± standard deviation (SD). For miRNAs microarray analysis, significant differentially expressed miRNAs were identified through Volcano Plot filtering: fold Change ≥2.0 and *t*-test *p*-value < 0.05. Hierarchical clusters were generated using MEV software (v4.6, TIGR). Unpaired or paired t-tests were performed for two groups’ continuous data comparison and one-way analysis of variance (ANOVA) for multiple groups’ continuous data comparison. Correlations between groups were assessed using Pearson’s test. *p*-values of < 0.05 were considered significant.

## 3. Results

### 3.1. Characteristics of Participants

Thirty patients were enrolled. Twenty-five (83.3%) of these patients achieved remission after EEN treatment and underwent a secondary ileocolonscopy, and the other five patients were excluded from this study. The mean duration of EEN was 5.4 ± 1.56 weeks. Ten healthy individuals undergoing screening ileocolonoscopies were also recruited. Samples of six patients and six healthy controls were used for the microarray analysis, while all samples were used for the qRT-PCR validation. Characteristics of participants are summarized in Table 2. CD patients were aged from 21 to 39 with the same disease behavior. Nineteen of them had ileocolonic CD while six were diagnosed as ileal CD. Ten patients received sulfasalazine or mesalazine treatment. After EEN therapy, CDAI scores and CRP levels reduced significantly in CD patients.

As miRNAs expression levels in microarray were variable among samples, in order to reduce false/erroneous candidate miRNAs, relatively higher stringency criteria were used: (1) background corrected intensities ≥200 in all samples; (2) fold change ≥2.0; and (3) *t*-test *p*-value of < 0.05. Some miRNAs were then selected for validation.

### 3.2. Alteration of MiRNAs Expression Profiles in Terminal Ileal Mucosa of CD after EEN Treatment (EEN-M)

#### 3.2.1. Comparison of miRNAs Expression Profiles in EEN-M and Inflamed Mucosa of Active CD

**Figure 1:**
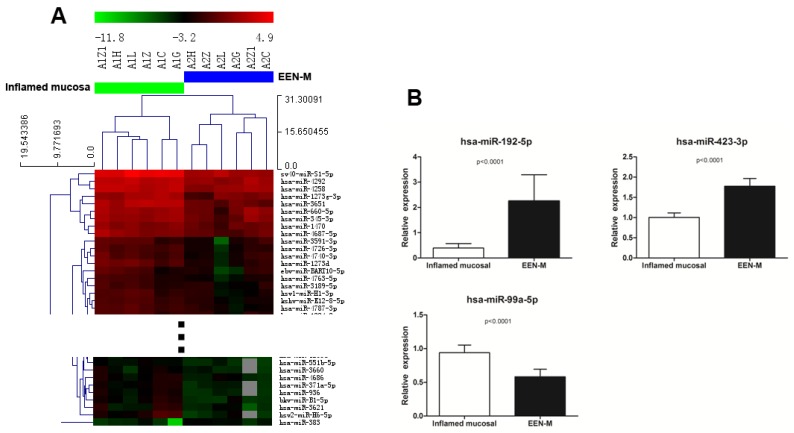
Comparison between inflamed mucosa of active CD and EEN-M: (**A**) heat map of differentially expressed miRNAs clearly separated inflamed mucosa and EEN-M; and (**B**) qRT-PCR validation of miRNAs differentially expressed in EEN-M. EEN-M, mucosa after exclusive enteral nutrition treatment.

Results of microarrays demonstrated the miRNAs expression profile of EEN-M was significantly changed compared with the inflamed mucosa before EEN, with the largest number of differentially expressed miRNAs in all comparisons. Inflamed mucosa were clustered together in a separate cluster from EEN-M. Of the 216 miRNAs exhibited significant differential expression (46 increased and 170 decreased in EEN-M), 30 miRNAs fit the stringent criteria with four up-regulated and 26 down-regulated in EEN-M. QRT-PCR on miRNAs (hsa-miR-192-5p, hsa-miR-423-3p, hsa-miR-199b-5p, and hsa-miR-99a-5p) showed that hsa-miR-192-5p and hsa-miR-423-3p had a significant expression increase in EEN-M, while the expression of hsa-miR-99a-5p was decreased (Figure 1).

#### 3.2.2. Comparison of miRNAs Expression Profiles in EEN-M and Non-inflamed Mucosa of Active CD

**Figure 2:**
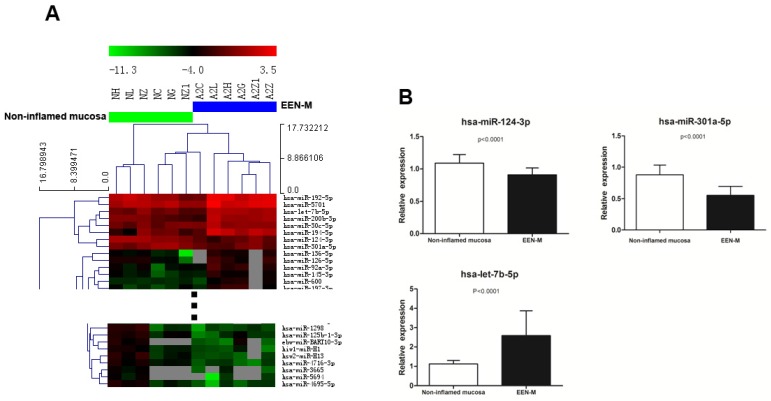
Comparison between non-inflamed mucosa of active CD and EEN-M: (**A**) heat map of differentially expressed miRNAs could not separated non-inflamed mucosa of active CD and EEN-M; and (**B**) qRT-PCR validation of miRNAs differentially expressed in EEN-M.

We then compared EEN-M and non-inflamed mucosa before EEN, and microarrays could not separate the tissue samples in the two groups, but there were still 73 miRNAs (28 up-regulated and 45 down-regulated in EEN-M) that exhibited significant differential expression between these two groups. Among the nine miRNAs that met the higher stringency criteria, differential expressions of hsa-let-7b-5p, hsa-miR-124-3p and hsa-miR-301a-5p were confirmed by qRT-PCR, while no significant difference was found in hsa-miR-192-5p and hsa-miR-31-5p (Figure 2).

#### 3.2.3. Comparison of miRNAs Expression Profiles in EEN-M and Healthy Controls

**Figure 3:**
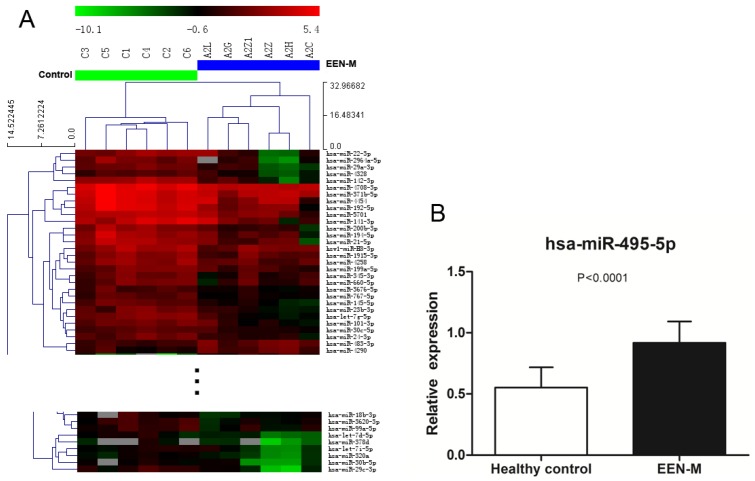
Comparison between EEN-M and healthy control: (**A**) heat map of differentially expressed miRNAs clearly separated EEN-M and normal samples; and (**B**) qRT-PCR validation of miRNAs differentially expressed in EEN-M.

When EEN-M and healthy controls were compared, significance analysis of differential miRNAs expression and hierarchical clustering analysis could also clearly separate EEN-M from the healthy controls, with 77 in a higher level and 70 in a lower level in EEN-M. Seventeen up-regulated and 30 down-regulated miRNAs fit the higher stringency criteria. Validation on five miRNAs (hsa-miR-630, hsa-let-7a-2-3p, hsa-miR-192-5p, hsa-miR-31-5p and hsa-miR-495-5p) confirmed hsa-miR-495-5p were differentially expressed (Figure 3).

**Figure 4:**
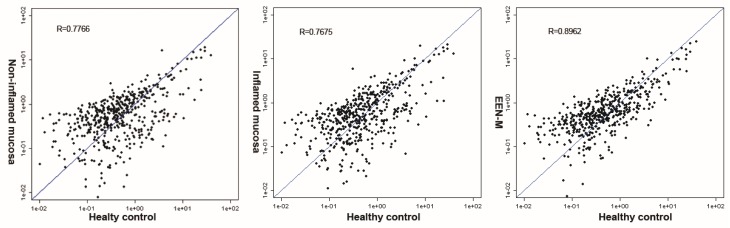
Correlation coefficient R-value and the scatter plot. The correlation coefficient R means the difference of miRNA samples. Bigger R values mean less difference.

Next, we investigated the similarity of miRNAs expression profiles between CD tissues and healthy controls using the correlation coefficient R-value and the scatter plot. The results showed that the expression profile in EEN-M group was most similar to those of the healthy controls (*R* = 0.8962) (Figure 4).

#### 3.2.4. Several miRNAs Expression in All Groups

**Figure 5:**
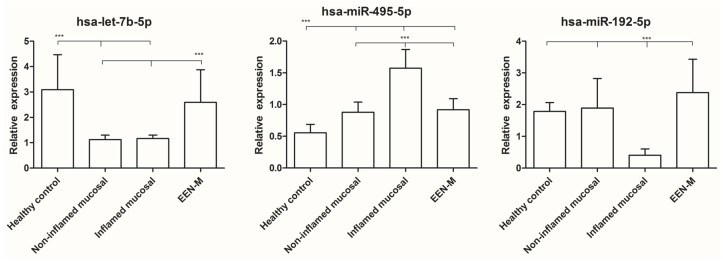
Expression of four miRNAs in all groups. *** *p* < 0.0001.

We also compared the expressions of hsa-miR-192-5p, hsa-miR-495-5p, hsa-miR-199-5p and hsa-let-7b-5p in all the four groups. One-way ANOVA showed no significantly different expression of hsa-miR-199-5p; hsa-let-7b-5p was decreased in both inflamed and non-inflamed mucosa of active CD as compared with healthy controls, but after EEN treatment, this differential expression disappeared; hsa-miR-495-5p was up-regulated in all CD groups, especially in inflamed mucosa before therapy; and hsa-miR-192-5p was down-regulated only in inflamed mucosa (Figure 5).

### 3.3. Correlation between miRNAs and CDAI and CRP before and after EEN

In order to ascertain possible correlation of miRNAs levels with CD activity parameters, Pearson’s correlation coefficient was used to evaluate the relationship between miRNAs (hsa-miR-192-5p, hsa-miR-423-3p, hsa-miR-99a-5p, hsa-miR-495-5p, and hsa-let-7b-5p) and CDAI and CRP. A significant correlation was detected between has-let-7b-5p and CRP both before and after EEN therapy (*r* = −0.518, *p* = 0.008, and *r* = −0.569, *p* = 0.003). However, neither CDAI nor CRP showed correlation with any other miRNA.

## 4. Discussion

In the present study, we focused on investigating mucosal miRNAs expression before and after EEN treatment for CD in adult patients. miRNAs are pivotal in the immune system, and changes in miRNAs expression were described in many immune-mediated diseases including CD [20,21]. Previous studies have described unique miRNAs expression profiles in inflamed and non-inflamed colonic mucosa as well as in the peripheral blood in active CD [22]. Terminal ileum, as the most commonly involved location, was also detected, and several miRNAs dysregulated in the inflamed and non-inflamed mucosa of active CD were identified [18,23]. These altered miRNAs may be a cause and/or a consequence of CD; hence, they may be affected by effective therapies and can be used as therapeutic targets and/or biomarkers. EEN is an effective treatment for the management of active CD. Patients with CD can benefit from EEN in terms of clinical remission, mucosal healing, improved nutrition status, and intestinal flora modification.

Our study indicated that in patients who achieved remission after EEN therapy, the miRNA expression profile was significantly altered, and it was more similar to the healthy one than the profile of non-inflamed mucosa before EEN. In addition, within the miRNAs verified by qRT-PCR, hsa-let-7b-5p was found to be significantly correlated with serum CRP level, both before and after EEN therapy.

In the present study, we also found that EEN therapy was effective to induce remission in adult CD patients, and the rate of remission was favorably with those reported previously [24,25]. As described in Materials and Methods, in the current study, only patients with mild-to-moderate active L1B1/L3B1 CD, who received no corticosteroids, immunosuppressive drugs, or biological therapy before were included. Besides, all smokers were excluded from this study. These restrictions may contributed to the high rate of remission in this study. In addition, an appropriate regimen for EEN and good compliance of our patients may also lead to the high response rate.

Several potential mechanisms of EEN have been raised. Recent evidence indicated that EEN could induce remission in CD by nutrients’ (like glutamine, vitamin D and short-chain fatty acids) direct anti-inflammatory effects, promoting restitution of the epithelial barrier, and affecting microbial composition [26]. Our recent study demonstrated that EEN could reduce remission through modifying mesenteric fat inflammation [27]. However, the exact mechanisms of EEN remains unclear.

There is accumulating evidence that miRNAs play an important role in CD pathogenic processes. For example, miR-141 is reduced in the epithelial cells of the inflamed colons from CD patients, which results in inflammatory cells trafficking into inflamed sites through miR141/CXCL12β pathway [28]; miR-122 can target NOD2 to decrease intestinal epithelial cell injury in CD [29]; miR-200b, which can partially protect intestinal epithelial cells from fibrogenesis in vitro, is dysregulated in the serum of the fibrosis CD group [30]; and miR-21 and miR-31 were indicated to be significant in IBD-associated carcinogenesis [31,32]. Among miRNAs of which alteration were confirmed by qRT-PCR in our study, some of them have also been reported to regulate inflammatory response in IBD. A recent study showed miR-495 could attenuate innate immune responses via suppression of NOD2 signaling pathway, and reduction of miR-124 could promote inflammation via IL-6/STAT3 pathway [33,34]. Increasing studies also indicated that diverse dietary factors have been found to interact with the genome, modify gene expression by affecting the expression profile of miRNAs or their function [35,36]. Davidson et al. reported that feeding rats with a diet containing *n*-3 polyunsaturated fatty acids could reverse the dysregulation of carcinogen-directed miRNAs, and Milenkovic et al. noted dietary polyphenols could modulate the expression of miRNA in the liver using mice model [37,38]. In the present study, we also observed a trend for normalizing of the miRNA expression after EEN therapy in patients with CD. These data suggest that EEN-induced remission may be associated with modification of miRNAs expression in the mucosa of CD, although more evidences are needed to confirm this hypothesis.

The uses of miRNAs are not only as potential therapeutic targets, but also as latent biomarkers. Both serum and mucosal miRNAs expression profiles can help in distinguishing CD from ulcerative colitis [22]. Let-7d and let-7e might be potential therapeutic biomarkers, which can predict the therapeutic effect of infliximab in CD [39]. Our study indicated that the decreased expression of has-let-7b-5p existed not only in inflamed but also in non-inflamed mucosa of active CD, and hsa-let-7b-5p was significantly correlated with serum CRP levels which is a commonly used inflammatory marker in CD. Thus, it may be a potential biomarker of inflammation in CD, which may be helpful to detect the mucosal inflammation in an early stage.

We applied stringent selection criteria to try to pick out more reliable candidate miRNAs. Among miRNAs fit this criteria, expressions of 30 miRNAs were commonly altered in both non-inflamed mucosa before EEN and EEN-M such as hsa-miR-495-5p, which may be important for the sensitization of the non-inflamed mucosa to the intestinal microbes and/or relapse of inflammation. Besides, differentially expressed miRNAs between inflamed mucosa and EEN-M may contribute to the mechanism of action of EEN in inducing disease remission in CD, and also may be potential biomarkers of CD activity. There were also numerous distinctively expressed miRNAs between non-inflamed mucosa and EEN-M, and the expression level were more similar to the healthy subjects after EEN treatment, such as hsa-let-7b-5p, which may account for the protective effect of EEN in CD.

There are several limitations of this study. First, the sample size is small, which may reduce the power of this study. Second, we only investigate the responders to EEN. It may be helpful to determine whether non-responders are characterized by a distinct miRNAs expression. Third, the results of the present study are characteristic for only the non-stricturing, non-penetrating luminal ileal/ileocolonic CD. The influence of EEN on miRNAs expression in other CD patients is not clear. Fourth, the age of control group was older than patients, which may influence the accuracy of healthy miRNAs profiles. Fifth, this was a single IBD center study, and only Han Chinese patients were included.

## 5. Conclusions

In conclusion, our data showed that in patients who achieved remission after EEN treatment, there was a trend for normalizing of the mucosal miRNAs expression profile. Besides, the expressions of mucosal hsa-let-7b-5p was correlated with serum CRP levels, both before and after EEN treatment. However, further multi-center studies with a larger number of patients are needed to confirm our results.

## Figures and Tables

**Table 1:** Primers used for quantitative real-time PCR.

Name	Primer
hsa-miR-21-5p	GSP:5′GGGGGGTAGCTTATCAGACTG3′ R: 5′GTGCGTGTCGTGGAGTCG3′
hsa-miR-31-5p	GSP:5′ GGGAGGCAAGATGCTGGC3′ R:5′GTGCGTGTCGTGGAGTCG3′
hsa-miR-192-5p	GSP:5′GGGGCTGACCTATGAATTG3′ R:5′CAGTGCGTGTCGTGGAGT3′
hsa-miR-423-3p	GSP:5′TAAGCTCGGTCTGAGGC3′ R:5′CAGTGCGTGTCGTGGA3′
hsa-let-7b-5p	GSP:5′GGGGTGAGGTAGTAGGTTG3′ R:5′CAGTGCGTGTCGTGGA3′
hsa-let-7a-2-3p	GSP:5′GCCCCTGTACAGCCTCCTA3′ R: 5′GTGCGTGTCGTGGAGTCG3′
hsa-miR-495-5p	GSP:5′GGGGAAGTTGCCCATGTTA3′ R: 5′GTGCGTGTCGTGGAGTCG3′
hsa-miR-199b-5p	GSP:5′GGGACCCCAGTGTTTAGACTAT3′ R: 5′GTGCGTGTCGTGGAGTCG3′
hsa-miR-124-3p	GSP:5′GGGTAAGGCACGCGGT3′ R: 5′GTGCGTGTCGTGGAGTCG3′
hsa-miR-301a-5p	GSP:5′GGGGGCTCTGACTTTATTGC3′ R: 5′GTGCGTGTCGTGGAGTCG3′
hsa-miR-99a-5p	GSP:5′GCCAACCCGTAGATCCGAT3′ R: 5′GTGCGTGTCGTGGAGTCG3′
U6	F:5′GCTTCGGCAGCACATATACTAAAAT3′ R:5′CGCTTCACGAATTTGCGTGTCAT3′

GSR, gene special primer; R, reverse; F, forward.

**Table 2:** Characteristics of all participants.

	CD Patients *N* = 25	Controls *N* = 10
Male/Female	17/8	4/6
Age (years)	27 (4.4)	42 (10.1)
Duration of disease (months)	14.6 (7.7)	n.a
Location		
ileum	6	n.a
ileocolon	19	n.a
Behavior (non-stricturing, non-penetrating)	25	n.a
CDAI before EEN	269.4 (55.5)	n.a
CRP before EEN	33.2 (18.5)	1.6 (1.3)
CDAI after EEN	77.7 (20.6) ***	n.a
CRP after EEN	3.6 (2.4) ***	n.a
Medication (sulfasalazine or mesalazine)	10	0
Smoker	0	0

*** *p* < 0.0001 compared with parameter before EEN.

## Supplementary Materials

The following are available online at http://www.mdpi.com/2072-6643/8/8/519/s1, Table S1: Comparison between inflamed mucosa of active CD and EEN-M. Differentially expressed miRNAs satisfying the higher stringency criteria. Table S2: Comparison between non-inflamed mucosa of active CD and EEN-M. Differentially expressed miRNAs satisfying the higher stringency criteria. Table S3: Comparison between EEN-M and healthy control. Differentially expressed miRNAs satisfying the higher stringency criteria in EEN-M (miRNAs in red color were also dysregulated in non-inflamed mucosa of active CD vs. healthy control).
