# Supplementary material for: Mucosal MicroRNAs Expression Profiles before and after Exclusive Enteral Nutrition Therapy in Adult Patients with Crohn’s Disease
Source: Nutrients. 2016 Aug 22;8(8):519. doi: 10.3390/nu8080519 (PMC4997431; doi:10.3390/nu8080519)
Supplement: Supplementary file 1 [file nutrients-08-00519-s001.docx]

Supplementary Materials: Mucosal MicroRNAs Expression Profiles before and after Exclusive Enteral Nutrition Therapy in Adult Patients with Crohn’s Disease

Zhen Guo, Jianfeng Gong, Yi Li, Lili Gu, Lei Cao, Zhiming Wang, Weiming Zhu and Jieshou Li

**Table S1.** Comparison between inflamed mucosa of active CD and EEN-M. Differentially expressed miRNAs satisfying the higher stringency criteria.

| **Up-Regulated in EEN-M** | | | **Down-Regulated in EEN-M** | | |
| --- | --- | --- | --- | --- | --- |
| miRNA | fold change | *p* value | miRNA | fold change | *p* value |
| hsa-let-7b-5p | 2.3861826 | 0.038728 | hsa-miR-345-3p | 0.4719807 | 0.01024 |
| hsa-let-7d-3p | 2.0428916 | 0.023975 | hsa-miR-4687-5p | 0.3284131 | 0.014588 |
| hsa-miR-423-3p | 2.6388662 | 0.01792 | hsa-miR-3651 | 0.1810143 | 1.88E-05 |
| hsa-miR-192-5p | 4.688249 | 0.006384 | hsa-miR-4733-3p | 0.4137229 | 0.00183 |
|  |  |  | hsa-miR-4287 | 0.4821401 | 0.018126 |
|  |  |  | hsa-miR-941 | 0.2656765 | 0.011081 |
|  |  |  | hsa-miR-4755-5p | 0.3084321 | 0.018564 |
|  |  |  | hsa-miR-4747-3p | 0.2418629 | 0.01695 |
|  |  |  | hsa-miR-3591-3p | 0.3308775 | 0.0092 |
|  |  |  | hsa-miR-4667-3p | 0.2882166 | 0.023994 |
|  |  |  | hsa-miR-4787-3p | 0.3078138 | 0.00057 |
|  |  |  | hsa-miR-3679-3p | 0.2118231 | 0.017204 |
|  |  |  | hsa-miR-4292 | 0.464667 | 0.000334 |
|  |  |  | hsa-miR-3620-3p | 0.3736027 | 0.011947 |
|  |  |  | hsa-miR-4449 | 0.4168009 | 6.28E-05 |
|  |  |  | hsa-miR-4749-3p | 0.2394813 | 0.000126 |
|  |  |  | hsa-miR-4469 | 0.4895157 | 0.044225 |
|  |  |  | hsa-miR-18b-3p | 0.2733701 | 0.005706 |
|  |  |  | hsa-miR-3162-3p | 0.4636114 | 0.00027 |
|  |  |  | hsa-miR-660-5p | 0.4923642 | 0.019759 |
|  |  |  | hsa-miR-99a-5p | 0.3925434 | 0.015456 |
|  |  |  | hsa-miR-4258 | 0.4286034 | 0.000336 |
|  |  |  | hsa-miR-3158-5p | 0.4867184 | 0.009247 |
|  |  |  | hsa-miR-3189-3p | 0.2712076 | 0.000434 |
|  |  |  | hsa-miR-361-3p | 0.2267635 | 0.001892 |
|  |  |  | hsa-miR-1273g-3p | 0.3440492 | 0.001628 |

**Table S2.** Comparison between non-inflamed mucosa of active CD and EEN-M. Differentially expressed miRNAs satisfying the higher stringency criteria.

| **Up-Regulated in EEN-M** | | | **Down-Regulated in EEN-M** | | |
| --- | --- | --- | --- | --- | --- |
| miRNA | fold change | *p* value | miRNA | fold change | *p* value |
| hsa-miR-31-5p | 4.598657126 | 0.013341 | hsa-miR-124-3p | 0.31706387 | 0.002511 |
| hsa-let-7b-5p | 2.692103037 | 0.011950 | hsa-miR-301a-5p | 0.469364294 | 0.034508 |
| hsa-miR-200b-3p | 2.575067962 | 0.041929 |  |  |  |
| hsa-miR-10a-5p | 2.579905844 | 0.036836 |  |  |  |
| hsa-miR-30c-5p | 2.041152474 | 0.042765 |  |  |  |
| hsa-miR-192-5p | 2.479854184 | 0.033833 |  |  |  |
| hsa-miR-5701 | 2.478531279 | 0.035708 |  |  |  |

**Table S3.** Comparison between EEN-M and healthy control. Differentially expressed miRNAs satisfying the higher stringency criteria in EEN-M. (miRNAs in red color were also dysregulated in non-inflamed mucosa of active CD vs healthy control.

| **Up-Regulated in EEN-M** | |  | **Down-Regulated in EEN-M** | |  |
| --- | --- | --- | --- | --- | --- |
| miRNA | fold change | *p* value | miRNA | fold change | *p* value |
| hsa-miR-483-3p | 2.031735345 | 0.014523 | hsa-miR-31-5p | 0.493600648 | 0.029764 |
| hsa-miR-4290 | 2.296200932 | 0.025071 | hsa-miR-24-3p | 0.317492859 | 0.002110 |
| hsa-let-7a-2-3p | 2.481257743 | 0.018707 | hsa-miR-103a-3p | 0.488887619 | 0.002851 |
| hsa-miR-519e-3p | 3.264784436 | 0.004803 | hsa-miR-200b-3p | 0.214625355 | 0.004045 |
| hsa-miR-204-3p | 2.481539155 | 0.003204 | hsa-miR-345-3p | 0.399274930 | 0.011041 |
| hsa-miR-4531 | 2.947384168 | 0.000215 | hsa-miR-320b | 0.442870537 | 0.018133 |
| hsa-miR-4308 | 3.331857202 | 0.001625 | hsa-let-7g-5p | 0.184475008 | 0.000066 |
| hsa-miR-205-3p | 2.860113925 | 0.000425 | hsa-miR-146b-5p | 0.326934914 | 0.001068 |
| hsa-miR-495-5p | 2.147266244 | 0.042434 | hsa-miR-101-3p | 0.343061636 | 0.003541 |
| hsa-miR-630 | 2.544748950 | 0.000378 | hsa-miR-767-5p | 0.324778592 | 0.000567 |
